# Robust Three-Microphone Speech Source Localization Using Randomized Singular Value Decomposition

**DOI:** 10.1109/access.2021.3130180

**Published:** 2021-11-23

**Authors:** SERKAN TOKGOZ, ISSA M. S. PANAHI

**Affiliations:** Department of Electrical and Computer Engineering, The University of Texas at Dallas, Richardson, TX 75080, USA

**Keywords:** Hearing aid device, low SNR, non-uniform microphone arrays, randomized algorithm, real-time implementation, singular value decomposition, smartphone, speech source localization

## Abstract

Direction-of-arrival (DOA) estimation is a fundamental technique in array signal processing due to its wide applications in beamforming, speech enhancement and many other assistive speech processing technologies. In this paper, we devise a novel DOA technique based on randomized singular value decomposition (RSVD) to improve the performance of non-uniform non-linear microphone arrays (NUNLA). The accurate and efficient singular value decomposition of large data matrices is computationally challenging, and randomization provides an effective tool for performing matrix approximation, therefore, the developed DOA estimation utilizes a modified dictionary-based RSVD method for localizing single speech sources under low signal-to-noise ratios (SNR). Unlike previous methods developed for uniform linear microphone arrays, the proposed approach with L-shaped three microphone setup has no ‘left-right’ ambiguity. We present the performance of our proposed method in comparison to other techniques. The demonstrated experiments shows at-least 20% performance improvement using simulated data and 25% performance improvement using real data when compared with similar DoA estimation techniques for NUNLA. The proposed method exploits frame-based online time delay of arrival (TDOA) measurements which facilitates the proposed algorithm to run on real-time devices. We also show an efficient real-time implementation of the proposed method on a Pixel 3 Android smartphone using its built-in three microphones for hearing aid applications.

## I. INTRODUCTION

The World Health Organization (WHO) reported that approximately 466 million people worldwide have hearing loss, and 34 million of these are children [1]. It is also projected that one in ten people, which accounts for over 900 million, will have disabling hearing loss in near future. In the US, approximately 15% of adults report some difficulty hearing, while around 50% of adults who are older than 75 have a hearing impairment [2]. Though, only 28.8 million adults in the US could benefit from using hearing aids [2]. Hearing aid devices (HADs) and Cochlear Implants (CI) were specifically developed to compensate for the loss in audibility. The performance of such devices can achieve close to normal hearing performance in normal conditions. However, their performance is compromised in the real world noisy environment. This causes degraded performance of the speech processing pipeline in real-world conditions and discomfort to the Hearing Aid (HA) users.

Hearing aid manufacturers [3]–[5] and numerous researchers have developed efficient signal processing algorithms to advance the performance of HADs, such as noise suppression, speech enhancement [6]–[8], acoustic feedback cancellation (AFC) [9], [10], speech source localization and beamforming [11]–[13], and speech-speaker recognition [16], [17]. From the psychoacoustics point of view, speech perception can be improved notably with these algorithms in noisy environments. Most of the aforementioned studies state that improving the signal-to-noise ratio (SNR) of the received noisy speech leads to the enhancement of speech with high perceptual quality.

Localizing sound sources is an important ability in daily life since it helps speech perception in a noisy environment with spatial unmasking effects [18], [19]. The human auditory system is fairly well known for the localization of sounds, in which it uses inter-aural time differences (ITDs) and inter-aural level differences (ILDs) [20], [21]. Hearing impairment on source localization has been thoroughly investigated [22]–[24]. Improving the SNR while preserving the quality and intelligibility of desired speech for hearing impaired people may not have a ‘spatially natural’ outcome because hearing loss hinders the localization ability. For instance, in [25], they discuss that hearing-impaired people have localization difficulties which are proportional to the level of hearing impairment. HADs can be beneficial for sound source localization, but they are not necessarily designed with this function, perhaps due to the size and processing power limitations. In [22] and [24], it is shown that commercial HADs negatively affect speech source localization (SSL) performance. In group conversations, the person should be able to locate a new speaker instantaneously when another speaker talks, otherwise, they can miss the conversation. Therefore, SSL is a critical element for hearing impaired people in real-world noisy conditions, and either visual or voice indication can assist them. Moreover, the SSL information can enhance the SNR of the desired speaker’s speech for the listener [26].

Most HADs have limited computational power due to their size, battery, and processor. For this reason, they are not able to handle complex signal processing algorithms, which makes implementing complex algorithms impractical for advancing their performance. In addition, hearing aid manufacturers have commercialized external microphones in the form of auxiliary devices like necklaces, pens, and table microphones to improve HAD’s performance. Although, these devices are rarely used due to their limited power and high price. As an alternative approach, popular smartphones can be used either as stand-alone devices or together with the application of HADs to help hearing aid users [15]. Smartphones are ubiquitous and most people including those with hearing loss use it, therefore, it has no additional cost to the HAD user. Smartphones with multi-core processors can run complex signal processing algorithms in a cost-effective and efficient way. Therefore, smartphones can be used as an assistive platform to implement HAD signal processing algorithms to improve the perceptual experience of HAD users [13]–[15], [31], [43].

This work aims to analyze the non-uniform non-linear “L-shaped” arrays (NUNLA) of microphones; the built-in microphones that are already available on most modern smartphones. This paper presents a novel noise-robust DOA method using the L-shaped microphone array structure available on modern smartphones to improve the experience of HAD users under noisy conditions. Sound is often assumed to originate from only one dominant speaker in various noisy environments, such as meeting rooms, restaurants, classrooms, and lecture halls [27]. This assumption simplifies the SSL algorithms. Therefore, we locate the speech source with the highest energy by utilizing the sinusoidal modeling in [26] for short overlapping speech frames. In the proposed setup, the estimated DOA information can be shown through visual information displayed on the smartphone panel or assisted via voice by communicating with HADs. Then, HAD users can reorient his/her position for optimum hearing reception or the position of the smartphone to receive the maximum SNR in the direction of the speaker.

In this paper, an L-shaped NUNLA geometry that is closely and unequally spaced by inter-element distances is investigated to prove the advantages of the proposed method. The proposed method extends the method in [55] and improves the DOA angle estimation for different noise types. Thus, the proposed method has superior accuracy performance and lower computational complexity. The proposed method has no left-right ambiguity compared to other methods [14], [31]. Our contributions can be listed as follows:

- We propose a TDOA SSL algorithm using randomized singular value decomposition (RSVD) to localize single speech sources under very low SNR levels.
- We also introduce a single-feature based, unsupervised voice activity detector (VAD) [56] as our second contribution. This improves the robustness and reliability of the proposed algorithm for the non-stationary background noise types and non-diffused noise sources [48].
- The third contribution is the real-time implementation of the proposed method on Android-based smartphones using only their built-in microphones and no external or additional hardware. Objective test results show that the proposed DOA estimation method finds the source direction with high accuracy.

The remainder of the paper is organized as follows. In section II, we review the works related to this research topic. In Section III, the SSL with respect to hearing aid (HA) applications is explained, and a brief description of left-right ambiguity and spatial aliasing is given. Section IV presents the proposed source localization method, and Section V analyzes the experimental results. Also, the performance of the proposed method is compared with other methods, and an explanation of the real-time implementation on Android smartphones is included in Section V. Last, Section VI concludes the paper.

## II. RELATED WORKS

Several approaches have been investigated for SSL to improve speech perception for hearing aids over the last decades. Popular methods can be categorized as: time delay of arrival (TDOA) methods [28]–[31], decomposing the auto-correlation matrix into signal and noise subspace [32]–[36], computing the steered response power to estimate DOA [37]–[40], using maximum likelihood(ML) [41], using sparse signal reconstruction [42] and deep learning based methods [43]–[47]. The deep learning based methods use the data-driven approaches trained on a large dataset to compute the DOA for single/multiple sources. These methods treat the DOA estimation problem as a ‘regression’ or ‘classification’ problem and use extensive training data to obtain estimation from deep-learning models. The drawback is that these methods require training and testing data to be hardware-matched for reliable real-time implementation. Although, there are many more varieties and variations of DOA estimation techniques, the above mentioned classification describes majority of the DOA estimation algorithms relevant to the current work. A comprehensive study of the state-of-art SSL algorithms can be found in [48]–[51]. Additionally, a summary of the recent works can be found in Table 1.

As stated earlier, SSL serves as an essential pre-processing technique that can be utilized to improve the SNR, suppression of background noise, and speech enhancement with good perceptual quality. Finding the direction of arrival (DOA) of the source signal by using a microphone array and beamforming is a popular approach for SSL. There are many factors that each affect the performance of this approach such as the type and geometry of the microphone array, the type of noise, the number of microphones, and the SNR level. Depending on requirements, there are infinite possible geometries and arrangements of microphone arrays. Over the years, more attention has been drawn to uniform linear microphone arrays (ULAs) and non-uniform linear microphone arrays (NULAs), whereas few studies have focused on the NUNLA [57]. Due to the infinite possible geometries, analyzing the NUNLA is generally complex, and yet prior methods [52]–[55] reported that it has significant advantages over ULA and NULA. Reference [52] presents a comprehensive overview of the use of a V-shaped microphone array structure, which is another geometry of NUNLA that uses a t-coil component to communicate with the HADs. The study suggests putting a microphone array on people’s necks, which signifies the performance of the NUNLA. Specifically, using it to reduce the acoustic feedback in HADs, shortening the reverberation, and improving the SNR by 10 dB relative to omni-directional background noise. In [53], a three microphone L-shaped geometry was proposed using TDOA estimates. They calculated the location of the source from the intersection of hyperbolic curves taken from the TDOA estimations. Another L-Shaped microphone array structure was suggested in [54] for impulsive acoustic source localization. This method focuses on a TDOA estimation technique that uses the orthogonal clustering algorithm. The method can work in reverberant environments at low sampling rates. In [55], ULA, NULA, and NUNLA(L-Shaped) geometries are investigated under the effects of low SNR. Current approaches have specific limitations, such as requiring large data lengths for sufficient operation, computationally too expensive, requiring a large number of microphones in the array, or poor performance under low SNR.

## III. SOUND SOURCE LOCALIZATION

Differences between captured signals from each microphone in the array produce inter-microphone time and level differences. This information can be effectively used in estimating the location of the source signal in the DOA algorithms. In order to process this information, there should be advanced signal processing algorithms to handle the data created by microphone arrays. For the current HADs, it is difficult to implement these algorithms due to device design limitations. In contrast, smartphones can coordinate with HADs by using their built-in L-shaped microphone arrays shown in Figure 2(b) with no external hardware, and carry out the high computational algorithms. Real-time DOA applications on the smartphone enable the HI individual to see the speech source location on the smartphone screen and focus their attention or re-orient the phone position to the desired speaker source. Re-orientation of the phone increases the SNR, thus improving speech enhancement performance and speech clarity.

### A. LEFT-RIGHT AMBIGUITY AND SPATIAL ALIASING

Left-right ambiguity is caused by the symmetry in microphone arrays using two microphones and it also depends on the spatial design of the microphone array and source location. This problem generally occurs in ULA and NULA structures due to the linear arrangement of the microphones in the array. Several microphone array configurations can solve the left-right ambiguity issue such as L-shape, circular, and spherical. In this work, the L-shape microphones array is chosen for the proposed method.

Spatial aliasing arises if the distance *d* between elements in a microphone array is not small to ‘spatially’ sample the sound waves [57]. Otherwise, DOA estimation will have ambiguities due to the undesirable peaks in the directivity pattern. Assuming the inter-element spacing of two microphones *d*, the time difference *τ* is denoted by (1) where *θ* is the estimated angle and the speed of sound *c* is assumed 343 m/s in the air.

(1) τ=dcosθ/c

Inter microphone distance *d* between microphones is given by:

(2) $$d\leq \frac{\lambda_{\text{min}}}{2}$$

where *λ*_min_ = *c*/*f*_*max*_ wavelength corresponding to the highest source frequency *f*_*max*_. For instance, the functional bandwidth of the source signal can be as much as *f*_*max*_ = 8.5 kHz if *d* = 2 cm is chosen and *c* is assumed *c* = 343 m/s. In general, the spatial distribution of the microphone arrays is fixed, which makes identifying the functional frequency bandwidth critical in accurately estimating the DOA.

**FIGURE 1.**
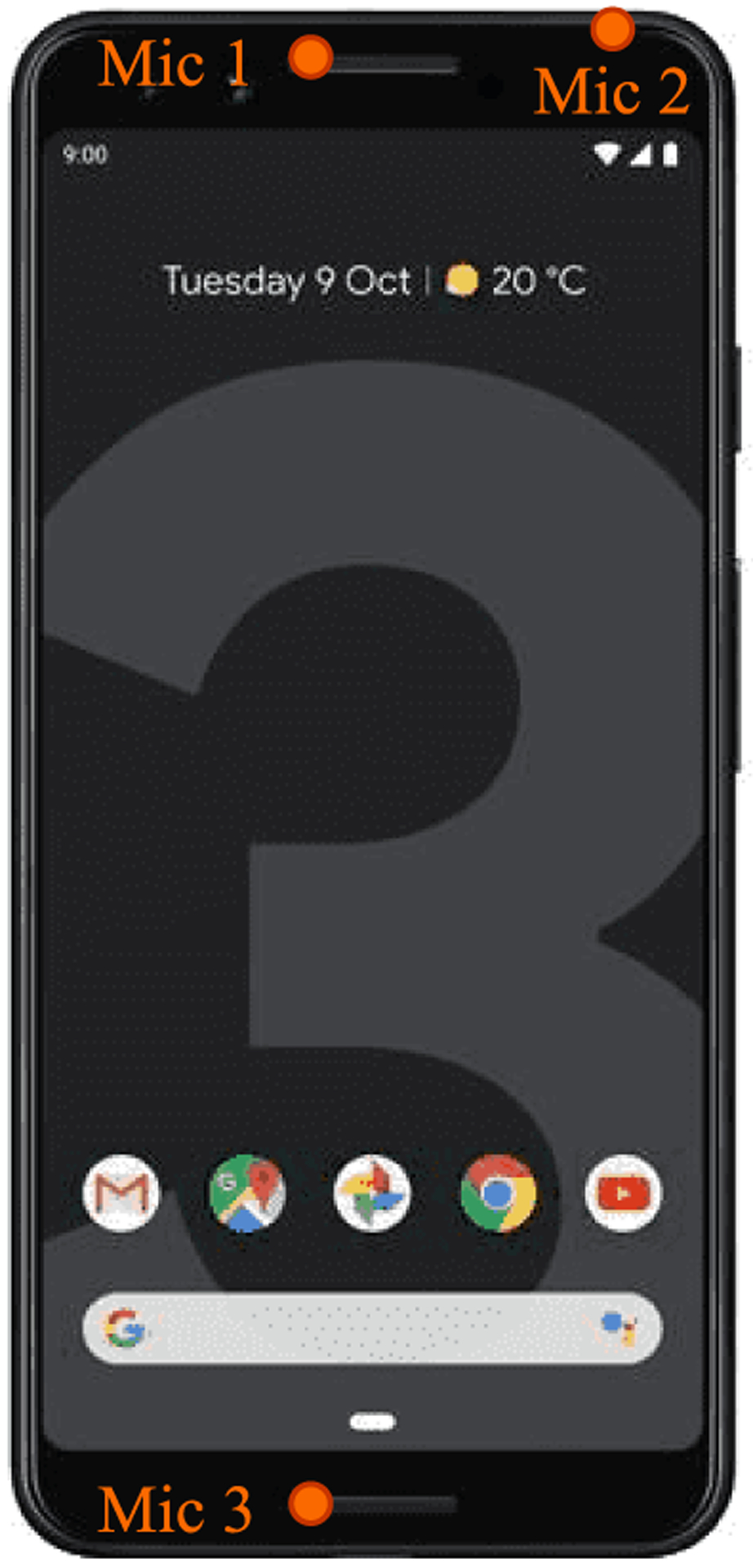
L-shaped 3 microphone array on Pixel 3 smartphone.

The positioning of the microphones in NUNLA architecture is not as linear as the previous case, which leads to different time delay between the microphones [57]. NUNLA architecture can provide more data and more precise SSL outcomes as compared to the ULA and NULA architectures. Depending on NUNLA orientation, it can handle a broader range of source frequencies than ULA. Additionally, NUNLA has an insignificant left-right ambiguity problem and less spatial aliasing [55]. Figure 1 shows a smartphone with three element NUNLA arranged in an ‘L’ shaped geometry.

## IV. PROPOSED METHOD

We use L-shaped three microphones, known as NUNLA, which is available on most modern smartphones. These microphones are located relatively close to each other as shown in Figure 1 so that they can contribute to the theoretical and practical aspects of our proposed method. Furthermore, our approach can be implemented on any other smartphones with three or more built-in microphones.

The goal of time-delay based DOA estimation is accurately finding the position of the desired source signal using microphone arrays with known geometry. All microphones are assumed to be theoretically identical to each other in this study. As stated previously, over-complete dictionary based randomized singular value decomposition (OD-RSVD) for SSL was developed. The premise of this algorithm is localizing the principal source and is similar to [33] and [36]. The proposed algorithm is computationally much lower compare to [42], [55], and performs better than [33], [55] under noisy conditions. Our approach is distinctly different from the previous SSL methods despite being inspired by some of their elements.

In this section, the signal model for DOA estimation is explained, and the algorithms used in the proposed method is detailed in the next sections. The general block diagram of the proposed method is shown in Figure 3, and a performance comparison is presented further in the paper.

### A. PROBLEM FORMULATION

Speech processing methods generally consider noisy speech *y*(*n*) as clean speech *s*(*n*) and additive noise *v*(*n*). We denote the signal model as:

(3) $$y_{i}(n)=s\left(n-\Delta \eta_{i}\right)+v(n)$$

where *y*_*i*_(*n*) is the noisy speech signal, and *i* = 1, 2, …, *K* for each *i*^*th*^ microphone. The received source signal at each *ith* microphone is expressed as *s* (*n* – Δ*η*_*i*_), and the time delay at each microphone is denoted as Δ*η*_*i*_. *v*(*n*) is the noise signal and is uncorrelated with the speech signal.

**FIGURE 2.**
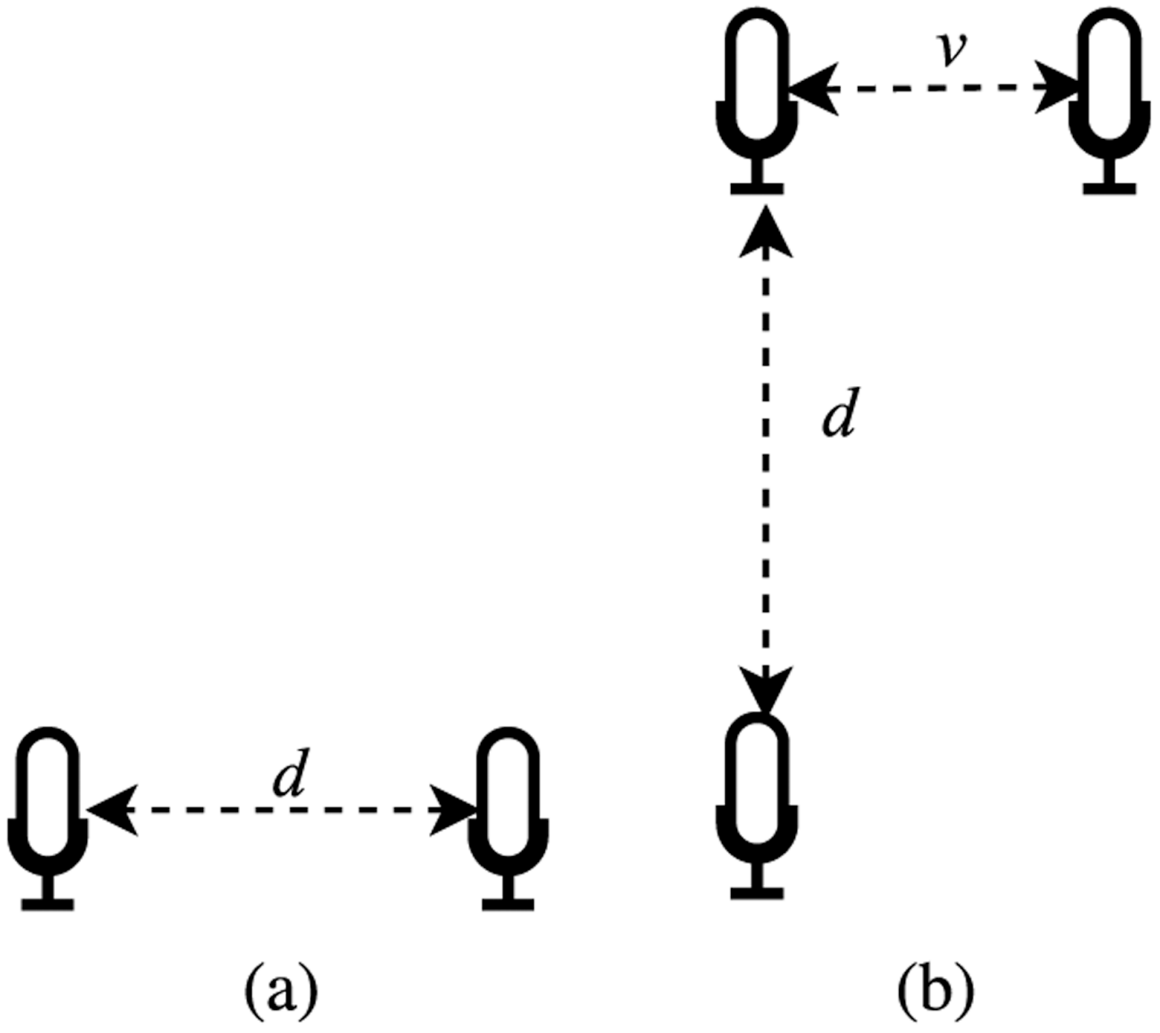
(a) Uniform linear arrays (ULA) and (b) Non-uniform non-linear arrays NUNLA where d and v are the inter-element microphone distances.

As demonstrated in Figure 2b, inter-microphone distances are denoted as d and v. The time difference Δ*t*_*ij*_ is given by:

(4) $$\Delta t_{12}=l\;\text{cos}(\alpha -\varphi )/\text{c}$$

(5) $$\Delta t_{13}=d\;\text{cos}\;\varphi /\text{c}$$

(6) $$\Delta t_{23}=v\;\text{sin}\;\varphi /\text{c}$$

where $\varphi ={\text{tan}}^{-1}\left(\frac{d}{v}\right)$, $l={\left(d^{2}+v^{2}\right)}^{1/2}$, and *c* is the known speed of sound. The values for the Pixel 3 smartphone are *v* = 2.8*cm*, *d* = 13*cm*, *l* = 13.29*cm*, and *φ* = 77.84°.

### B. DOA ESTIMATION

The estimation of DOA angle $\hat{\theta }$ assumes the following two conditions: the microphone array geometry and speed of sound (denoted as *c*) are known. The proposed DOA estimation algorithm has 2 main steps: sinusoidal modeling of speech using Auto-regressive (AR) model, and narrow-band DOA estimation using RSVD and over-complete dictionary matrix.

**FIGURE 3.**
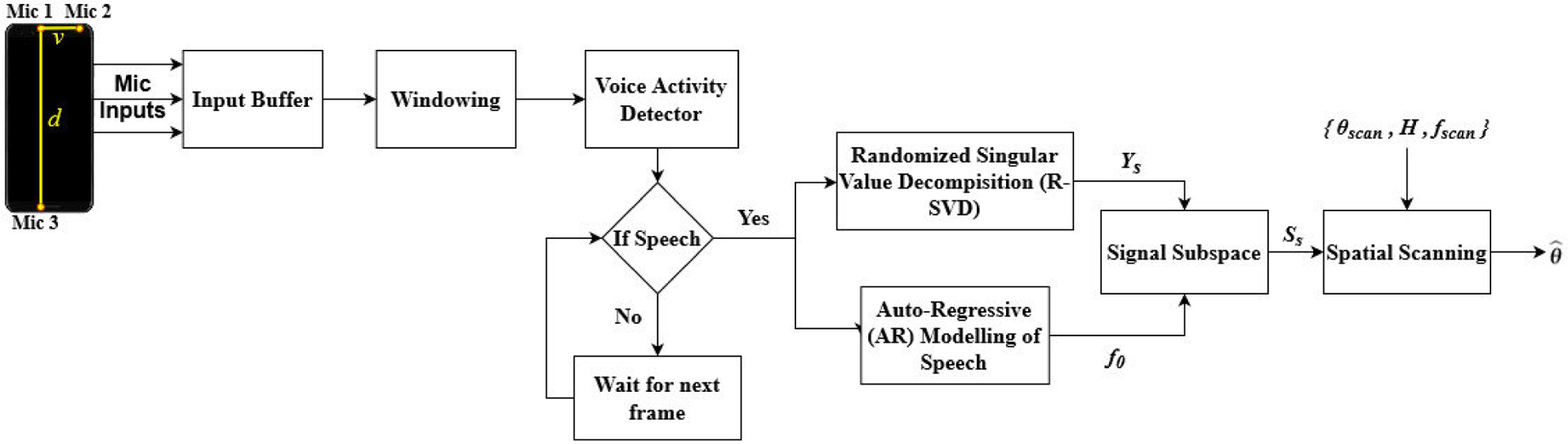
Block diagram of the real-time processing of the proposed DOA estimation method.

Figure 3 shows the general pipeline of the proposed method. First, the microphone inputs are framed, buffered, and Hamming window with 50% overlap is utilized to the signal. Next, VAD is utilized to classify the incoming frames as speech and noise. At the output of the VAD, we have the input speech frames *Y*_*i*_(*n*), *n* = 1, 2, …, *L* for each micro phone, *i* = 1, 2, 3 and *L* is the frame size. The speech frames will be fed into the RSVD and AR modelling of speech for further steps. In DOA estimation path, RSVD is performed to obtain the subspace of the signal at each microphone and using the over-complete dictionary matrix *H* the scanning is performed to estimate the DOA angle. The general procedure of the DOA estimation using OD-RSVD method is described in detail in Algorithm 1.

**FIGURE 4.**
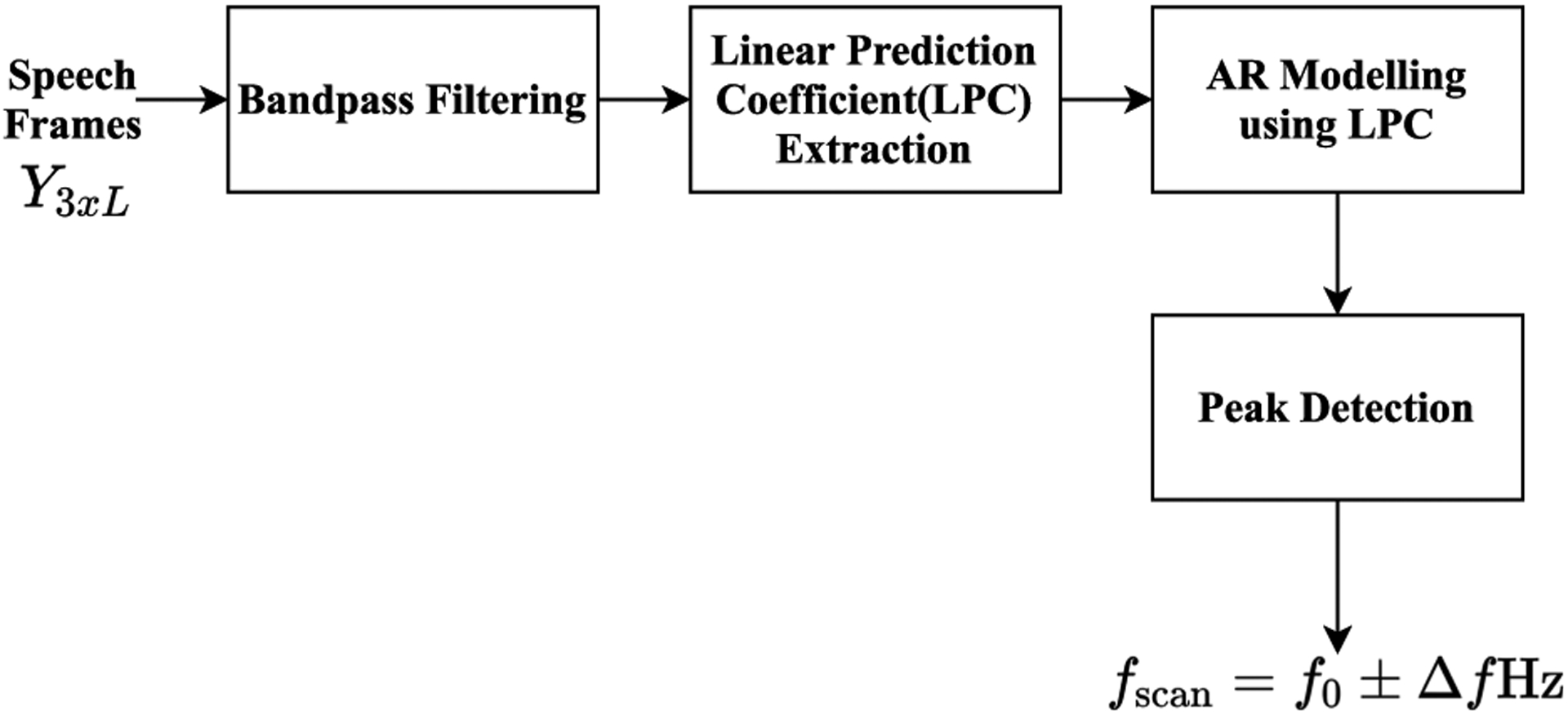
Block diagram of speech modeling to obtain *f*_*scan*_.

As shown in Figure 4, the steps are used to handle speech data before performing DOA estimation. First, band-pass filter is utilized between 300*Hz* and 3400*Hz* since smaller frequency bandwidth reduces the scanning complexity and also more speech content can be found in this range. This filter reduces bandwidth and avoids spatial aliasing caused by the distance between microphones [55]. Next, AR modeling is performed using the LPC coefficients to predict the sinusoidal peaks in each *k*^*th*^ frame. By utilizing this model for speech data, the dominant components of speech can be represented in noisy environments with exponentials [57]. These exponentials will be used for DOA estimation. Estimation of the dominant frequency, *f*_0_ in each frame can be found by peak point in the AR model frequency spectrum. The *f*_*scan*_, frequency vector scan, will be calculated by using *f*_0_. To decrease the computational complexity of the algorithm, the range of scanning frequency narrowed to *f*_*scan*_ = *f*_0_ ± Δ*f Hz*, Δ*f* = 200 *Hz*. A single speech source is used in the method because it is a non-stationary wideband signal. The broadband speech is transformed into a ‘dominant’ narrowband sinusoid. AR modeling using linear predictive coefficients is utilized to handle speech sources under low SNR [27].


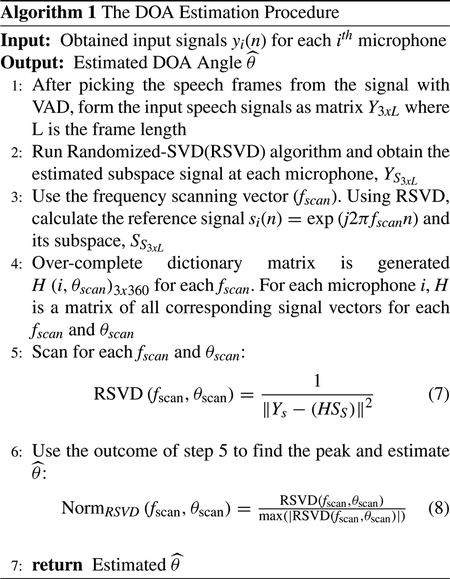


In Algorithm 1, when *θ*_*scan*_ meets the estimated angle $\hat{\theta }$ in (8), the result of (8) yields to maximal value(unity) for the far field scenario where *θ*_*scan*_ = *θ*_*start*_ *: θ*_*end*_. In (7), size of *Y*_*s*_ is 3 × 1, *H* is 3 × 1 and *S*_*S*_ is 1 × 1 for each iteration.

The *O*(*n*) time complexity for the proposed method is approximated as *O*(*L*^2^) with known *f*_0_ and *H*, where *L* is the frame size. There is a clear advantage of our approach in computational complexity as compared to [55].

### C. RANDOMIZED SINGULAR VALUE DECOMPOSITION

Randomness has occasionally surfaced in the numerical linear algebra literature. It is standard to initialize iterative algorithms for constructing invariant sub-spaces with a randomly chosen point. Random sampling can identify a subspace that captures most of the action of a matrix [58]. In various cases, this approach exceeds in terms of accuracy, speed, and robustness compared to classical methods [59]. There are several forms of approximation techniques based on the randomization idea. The method follows the pattern: re-processing the matrix, taking random samples from the matrix, post-processing the samples, and computing the final approximation.

The main assumption in this process is that the sources can be considered as point sources. By using this assumption, the underlying spatial spectrum will be sparse, and we can resolve this matter utilizing the randomized singular value decomposition (RSVD).


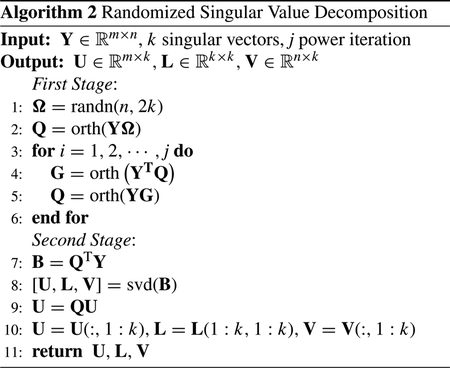


***Y*** is the speech frame an *m* × *n* matrix as input and *k* = 3 singular vectors. *j* is used to improve the accuracy of the approximation and generally chosen 1 or 2 [58]. *U* and *V* are the left and singular vectors, respectively. *L* is the diagonal matrix of singular values. **Ω** is *n* × 2*k* Gaussian i.i.d matrix.

At the first stage, a low dimensional subspace that approximates the column space of Y is constructed. After calculating the subspace’s orthogonal basis **Q**, we get an approximated SVD of *Y*. Then, regular SVD is performed on the small matrix B to get the approximated Y. The time complexity of the algorithm is approximately O(*mn* log(*k*)).

For this algorithm, the objective is to use random projection to identify the subspace of the signal capturing the dominant actions. This method helps the calculation of the near-optimal decomposition of Y.

### D. VOICE ACTIVITY DETECTOR

In real life, people are exposed to different types of noise, and the DOA estimation methods yield inaccurate decisions in the presence of background noise. The existence of noise leads to false peaks which indicates performance drops for subsequent speech processing blocks. Therefore, the VAD corrects the preliminary DOA and predicts $\hat{\theta }$ by differentiating noisy speech frames from only noise frames. As shown in Figure 3, if the current frame has non-speech data, the incoming frame does not pass through the system and the DOA result is retained from the previous frame; otherwise, the DOA estimate is updated as shown in (9):

(9) $${\hat{\hat{\theta }}}_{i}=\{\begin{array}{c} {\hat{\theta }}_{i-1},\;\text{if}\;VAD\;=\;0(\text{Noise})\\ {\hat{\theta }}_{i},\;\text{if}\;VAD\;=\;1(\text{Speech}) \end{array}$$

where ${\hat{\hat{\theta }}}_{i}$ represents revised DOA estimate for *i*^*th*^ frame. Consequently, the VAD tracks noise-only frames to smoothen the DOA estimation. A single feature-based is utilized to reduce the computational complexity for real-time operation. Spectral Flux (SF) feature-based VAD is preferred in our approach [56]. The SF feature is defined by (10):

(10) $$SF(k,i)=\frac{1}{N}\sum_{k}{\left(\left|X_{i}(k)\right|-\left|X_{i-1}(k)\right|\right)}^{2}$$

for *k*^*th*^ frequency bin and *i*^*th*^ frame, *k* = 1, 2*, .., N*. | ● | denotes the magnitude spectrum. A non-complex thresholding method is used, followed by a decision buffer, to reach a final VAD and is given by (11):

(11) $$VAD(i)=\{\begin{array}{c} 0\text{(Noise),}\;\text{if}\;SF(k,i)\;<\;\Delta \\ 1\text{(Speech)},\;\text{if}\;SF(k,i)\;\geq \;\Delta \end{array}$$

where Δ is the calibration threshold is calculated using cumulative averaging from the *T* initial frames. *T* determines how many frames are presumed as noise. The SF feature performs sufficiently under stationary noise conditions [56]. For non-stationary noise types, *D* is defined as a decision buffer and it is used for the VAD decision. The system waits for *D* consecutive frames until the VAD outputs as speech. Even though some delay is created in the output, VAD helps with stabilizing the DOA estimation. If the noise condition changes over time, the VAD will be re-calibrated, like previous VADs in [60].

## V. EXPERIMENTAL SETUP, RESULTS AND DISCUSSION

In this section, the obtained results of the proposed robust and faster DOA estimation method are presented. Several experiments are conducted to highlight the advantages of the proposed DOA estimation method for the NUNLA structure. The performance comparisons with similar methods [33], [37], [55] are also presented. To analyze the performance of the DOA methods, the average root mean square error (RMSE) is calculated. Lower RMSE values show better SSL performance.

(12) $$\text{RMSE}\left({}^{\circ}\right)=\sqrt{\frac{1}{N_{F}}\sum_{i=1}^{N_{F}}{\left(\theta_{i}-\hat{\theta_{i}}\right)}^{2}}$$

where $\left(\theta_{i}-\hat{\theta_{i}}\right)$ is the estimation error between correct DOA and the estimated DOA angle.

### A. SIMULATED DATA

The simulated data is produced using clean speech from TIMIT [61] and HINT [62] databases with additive noise. The noise files are collected outdoors with smartphones. The room impulse response (RIR) is simulated with Image-Source Model [63]. The resolution of the simulated dataset is set for 10 degrees. The sampling frequency is 16 kHz for the simulated data due to the databases, however, the higher sampling frequency can also be used depending on the application. Based on the fixed geometry of Pixel 3’s microphones, the distances between the microphones are *v* = 2.8*cm* and *d* = 13*cm*. The microphone array is assumed to be in the center of the room and the room size is 5*m* × 4*m* × 3*m* (*W* × *L* × *H*). The distance between the microphone array and the speaker is 1 meter. Noisy data is simulated with Machinery, Traffic, and Babble at three different SNRs, −5dB, 0dB, and 5dB. Approximately ten hours of noisy speech dataset for three-microphone is prepared for the simulated data.

### B. RECORDED DATA

Our goal is also implementing the proposed method on the smartphone for people’s hearing improvement, thus real recorded data is necessary to show the performance of the method. The data is recorded in a room approximately the same size that is used for the simulated data, and reverberation time is 300*ms* for the room. Loudspeakers are placed apart from each other so that the resolution is 20° for the real-time recording, and speaker distance from Pixel 3 is again 1 meter. Approximately, 36 minutes of audio data is recorded using speech files from TIMIT and HINT datasets. The sampling frequency is 48 kHz for the recorded data. For the noisy case, another loudspeaker, which is placed at the corner of the room, plays the noise files and the dataset is recorded with Pixel 3 smartphone for analysis with Machinery, Traffic, and Babble at three different SNRs, −5dB, 0dB and 5dB. These data files are available at [64] upon request.

### C. OBJECTIVE EVALUATION

The performance of the proposed method is evaluated using simulated and real recorded data. The comparisons are tested with the same dataset as the proposed method. The frame length *L* is 20*ms* in all evaluations. Firstly, we present results for the experiments using the simulated data. In addition, we present the computational processing time of the algorithm with different data lengths.

**FIGURE 5.**
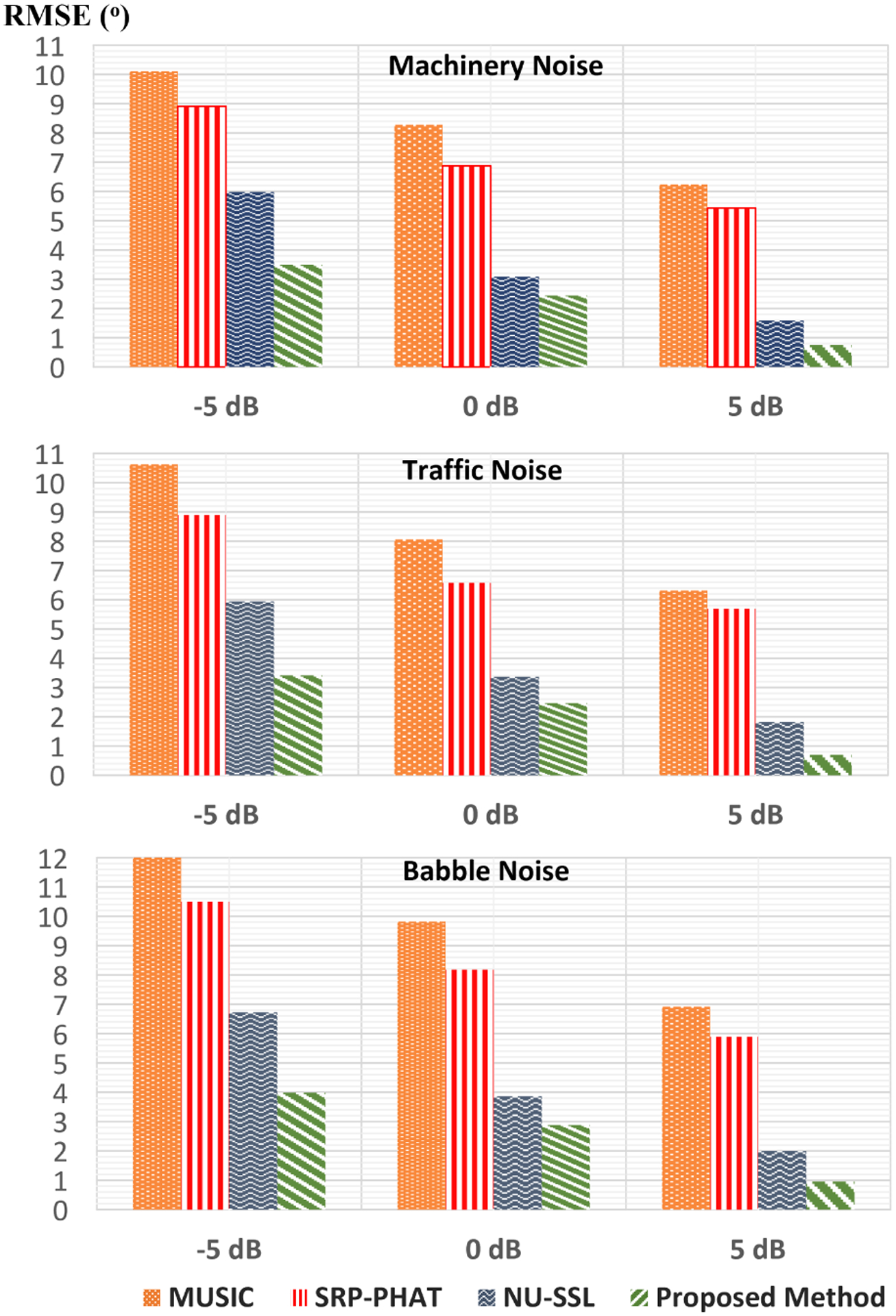
RMSE (°) results for DOA estimation using simulated data under machinery, traffic, and babble at −5dB, 0dB, and 5dB.

Our proposed method compared to the baseline methods such as [33], [37] and [55]. In [33], Multiple Signal Classification (MUSIC) based DOA algorithm is presented. In [37], a robust algorithm, Steered-Response Power Phase Trans-form (SRP-PHAT) is performed. In [55], the Singular Value Decomposition (SVD) based DOA algorithm is introduced. These methods are compared under Machinery, Traffic, and Babble at three different SNRs, −5dB, 0dB, and 5dB. Under high background noise, HAD users have difficulty understanding speech coming from a certain direction. To demonstrate this case, the SNR values are varied for the estimation of the DOA angle. The comparison of the proposed method to the other DOA methods using simulated data is illustrated in Figure 5. As it is seen from the figure, our proposed method performs at least 20% among all other methods under all conditions. Another observation is that the performance gap between the MUSIC and SRP-PHAT is less as SNR increases. Overall observation from the result is that the performance of all methods increases with increasing SNR.

**FIGURE 6.**
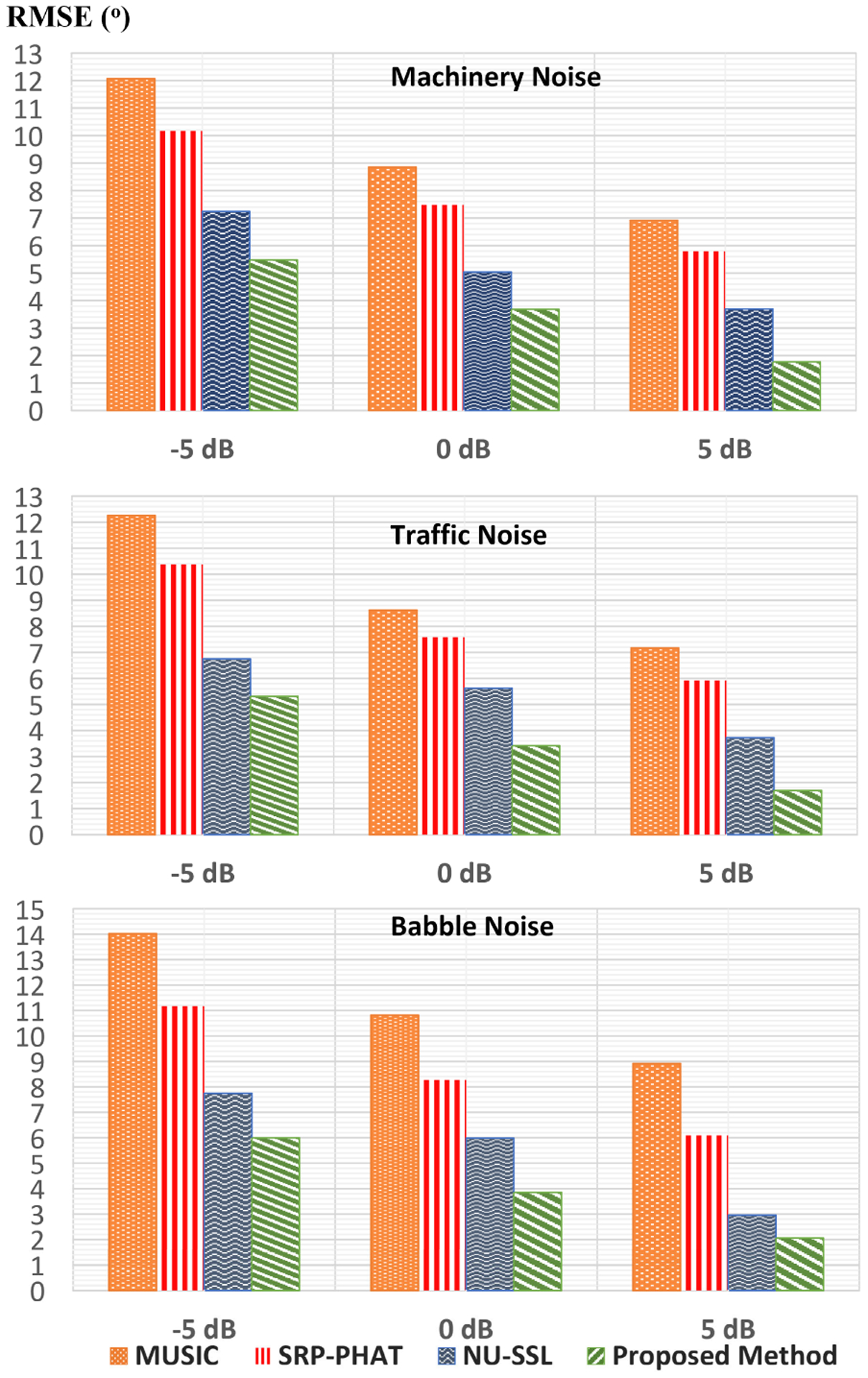
RMSE (°) results for DOA estimation using recorded data under machinery, traffic, and babble at −5dB, 0dB, and 5dB.

Figure 6 shows the comparison of the proposed method to the other two DOA methods using smartphone recorded data under Machinery, Traffic, and Babble at three different SNRs, −5dB, 0dB, and 5dB. As explained previously, the data was recorded by placing loudspeakers around the Pixel 3 smartphone with 20° resolution. Showing the real recorded data makes the proposed method more powerful to real-life noise and reverberation because the aim is to use this method in a real environment for HAD users. The proposed method shows a significant reduction in RMSE over all noisy conditions compared to the other methods. For recorded data, it can also be noted that the performance of all methods improves as SNR increases. The difference between results using the simulated and the recorded data can be observed from the objective measures. This variance can be caused by the three built-in microphones of the smartphone which can have different characteristics from each other and real-environment conditions. Overall, the results show that the proposed method is sufficient for real-world conditions. This proves that the application will be helpful as a visual indicator for HI people.

**FIGURE 7.**
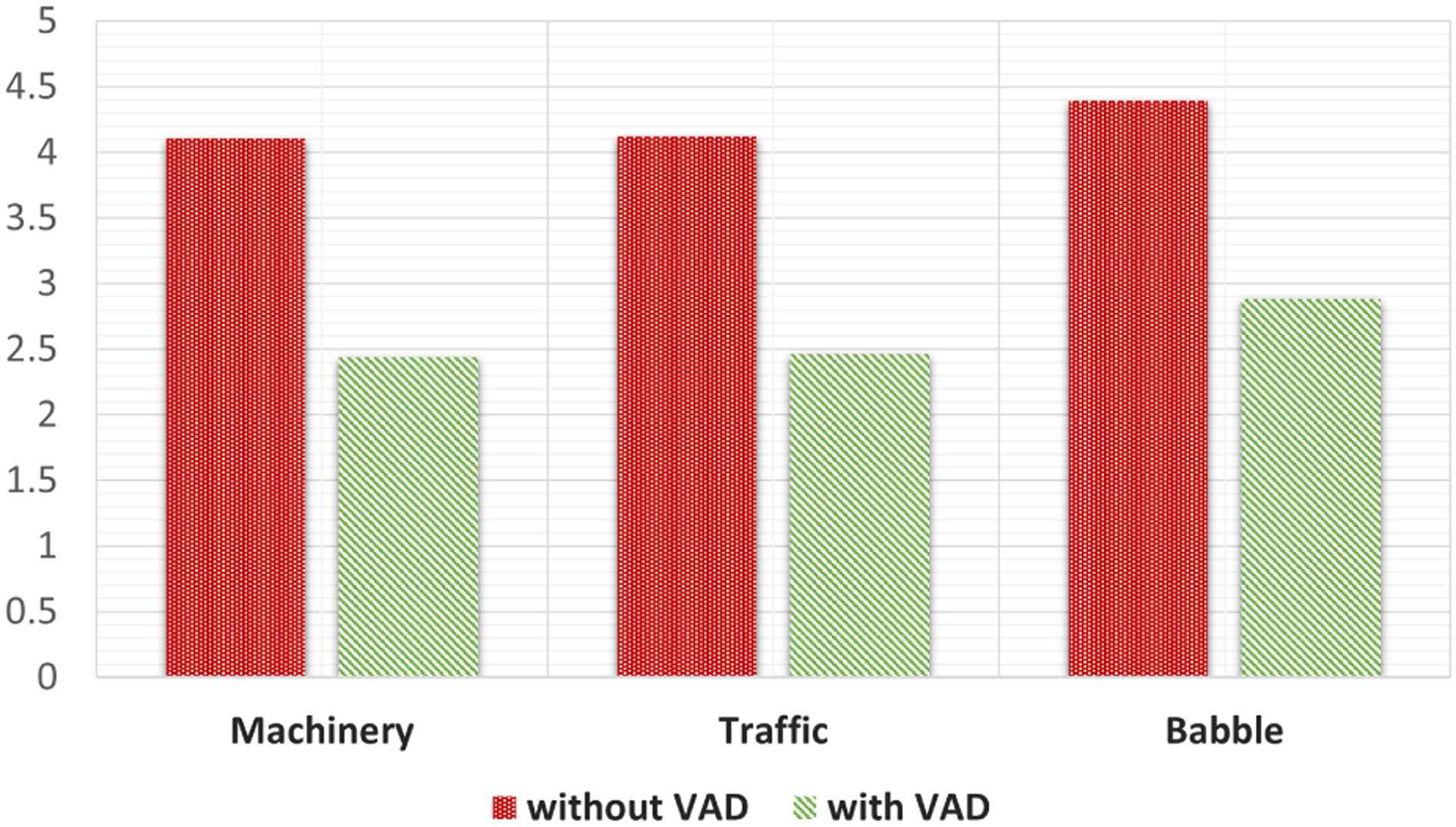
RMSE results for DOA estimation with and without VAD.

In proposed method, an unsupervised SF based VAD is employed to discriminate between speech and non-speech segments in the incoming audio frame. VAD plays a significant role in the reliability and robustness of the proposed DOA estimation algorithm for low SNR cases. Input signals from three microphones are processed by the VAD. If the input frame is speech then the VAD labels that frame as speech and the method estimates the DOA. If the input frame is determined as noise, the previously DOA estimation results will be used. Figure 7 depicts the effect of VAD in the proposed method at 0 dB SNR using simulated data, and this shows VAD has a positive effect on our method since it tracks noise-only frames to smoothen the DOA estimation.

Overall, the best results(lowest RMSE) are seen under machinery, and the worst results(highest RMSE) are under Babble noise as shown in Figures 5,6, and 7. This is caused by the stationary property of machinery noise, and the non-stationary property of babble noise due to its multiple speech characteristics. Since this work considers only using 3 microphones, the methods require more microphone for better performance.

To show the complexity of the proposed algorithm, we profiled the proposed method and compared it to other methods. Table 2 shows the processing times at different data lengths. In this table, audio frames at different data lengths are directly fed to the system, and actual time taken by the algorithm is provided. This evaluation has been done by profiling the method on MATLAB using a PC with i7-6700 CPU. The table shows that MUSIC and SRP-PHAT are not good candidates for real-time processing. The reason is MUSIC-based methods require performing online eigenvalue which adds a significant amount of computations and SRP-PHAT has excessive computation due to the grid search. Also, the table indicates that the processing times are less than the frame length of data for NU-SSL and the proposed method. Furthermore, the proposed method has the least processing time among all four methods which allows real-time implementation algorithm without compromising the accuracy of the method. Last, data length has a negative effect on the cost of deployment which means larger data length leads to higher computational time. Based on the processing times and average RMSE results for the proposed method, there is a trade-off due to the data length. There is an obvious performance improvement as the data length increases, as the algorithm has more data to work. For instance, RMSE values are 2.56°, 1.6°, and 0.7° for 20*ms*, 100*ms*, and 500*ms* in quiet room, respectively. Since the error for 20*ms* is adequate for the DOA estimation method and has a very low processing time, it is preferred for all objective evaluations and real-time implementation.

To evaluate the RMSE(°) results for certain different angles, we carried out simulations for the proposed method. Since the Figure 5 and 6 depicts average RMSE(°) for all angles, DOA estimation per angle has been done in Table 3 using real recorded data. Table 2 shows the performance evaluation of the proposed method with Babble noise at three different SNRs. Babble noise is chosen because this noise type generally has the lowest RMSE among others due to its complicated characteristics. In this objective evaluation, the real recorded data is used to show the real-environment performance of the proposed method. Due to the location of the built-in microphones on Pixel 3, there is a slight increase at 0° and 180°. We can see that the method performs in acceptable error levels for real-world conditions.

**FIGURE 8.**
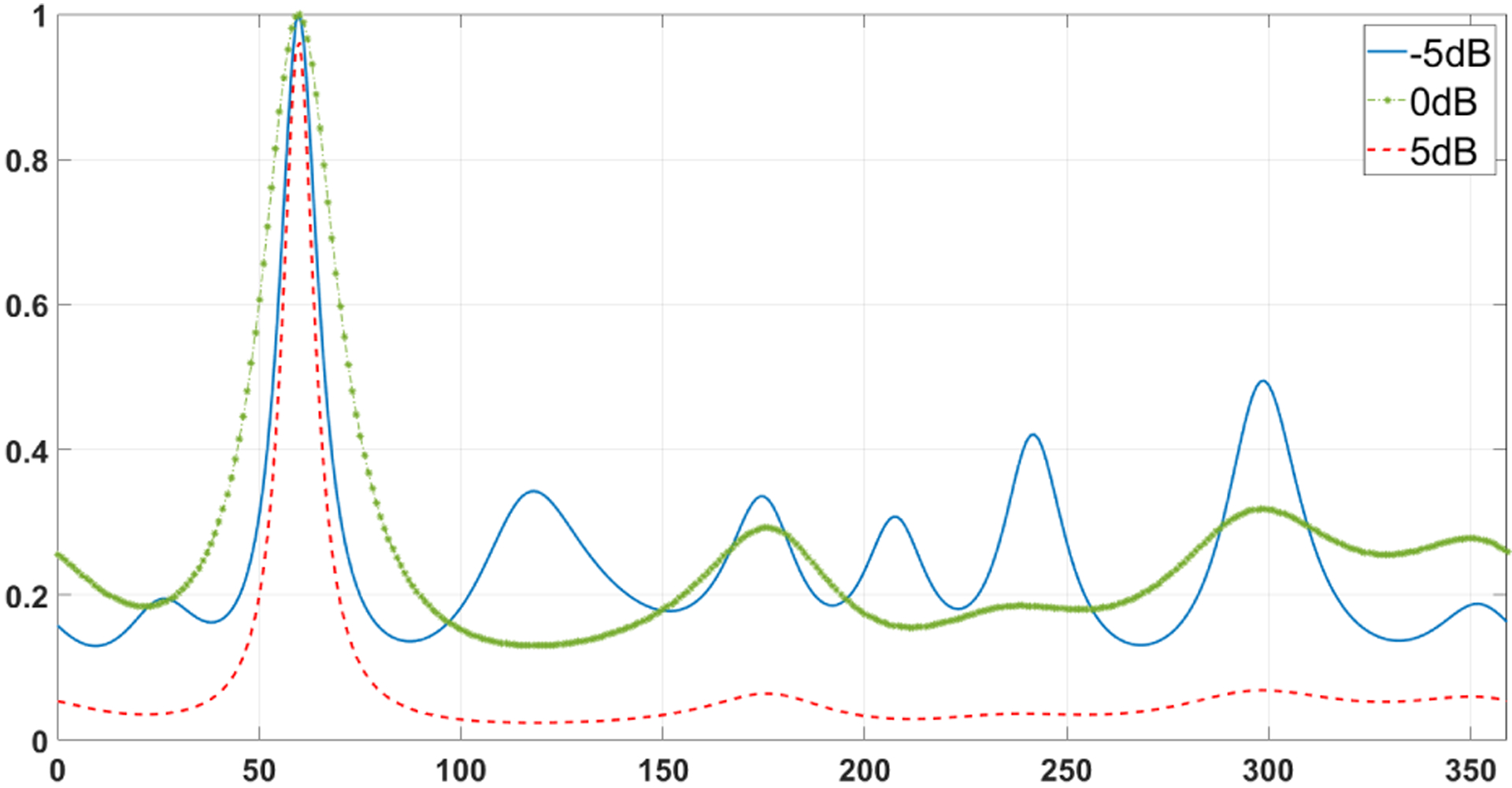
Linear directivity pattern (LDP) for the proposed method.

For further performance analysis, a linear directivity pattern (LDP) plot is used as another metric. Figure 8 shows the LDP of the source at 60° with babble noise with three different SNRs since the babble noise is the most challenging noise for the system. It can be seen that the decrease in SNR leads to a broader pattern in the plot. The DOA estimation errors can also be decreased by increasing SNR with the right orientation of the array to speaker location and performing proper pre-filtering method on the signal received at microphones. The figure indicates that there is no left-right ambiguity in the proposed method. Additionally, we can infer that when the SNR level is high, peaks that indicate spurious peaks are much lower. These errors can be referred to incorrect estimation of maxima in (8) inaccuracies due to the high presence of noise.

### D. REAL-TIME IMPLEMENTATION ON ANDROID BASED SMARTPHONE

In this work, our main goal is to present an especial three microphone array architecture shown in Figure 2(b) and its associated SSL method for real-time implementation on a smartphone with three built-in microphones as an assistive application for HAD users. In this section, the real-time implementation of the proposed algorithm is presented. Android operating system (OS) allows us to access the three built-in microphones of the smartphone. The proposed method is implemented on the Android Pixel 3 smartphone, however, the method can be implemented on most modern Android smartphones with 3 built-in microphones.

**FIGURE 9.**
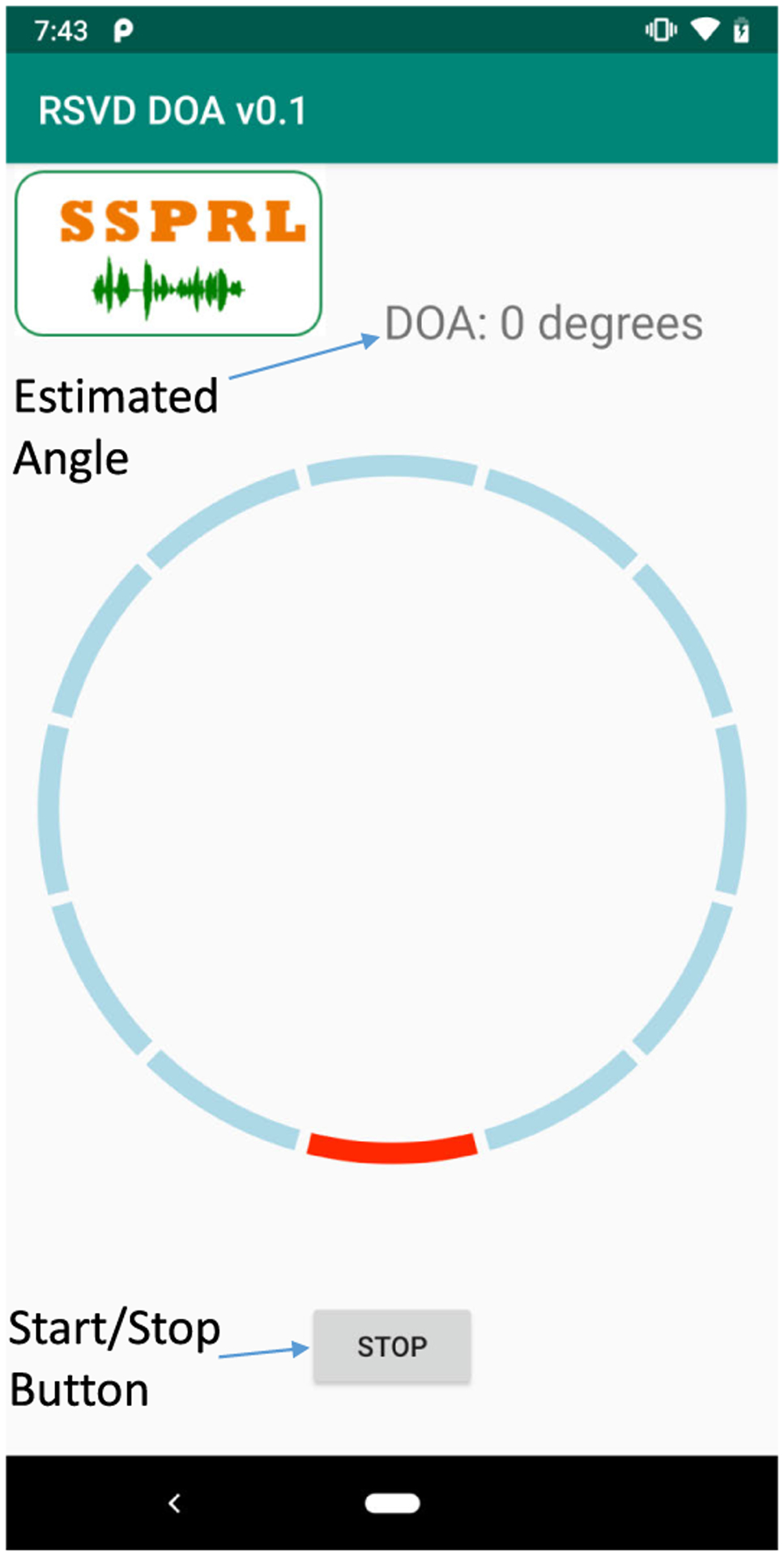
Screenshot of the developed application on android smartphone.

To achieve the lowest audio I/O latency on smartphones, the sampling rate of 48 kHz is required. This latency is related to the input/output of the smartphone. Therefore, a frame-based structure is used for real-time implementation with the frame size of 20ms and sampling frequency of 48 kHz. A snapshot of the developed application can be seen in Figure 9. When the button shows ‘START’, the application does not do any kind of signal processing. Switching the button on the touch screen of the smartphone enables the DOA algorithm to process the incoming audio frame by applying the proposed algorithm. The application displays the estimated DOA angle with a red marker and it shows the estimated angle on the top right of the app. If the incoming audio frame is estimated as not a speech, the marker points to the last estimated DOA location. The application has been pre-tuned to perform optimally under different noisy conditions.

**FIGURE 10.**
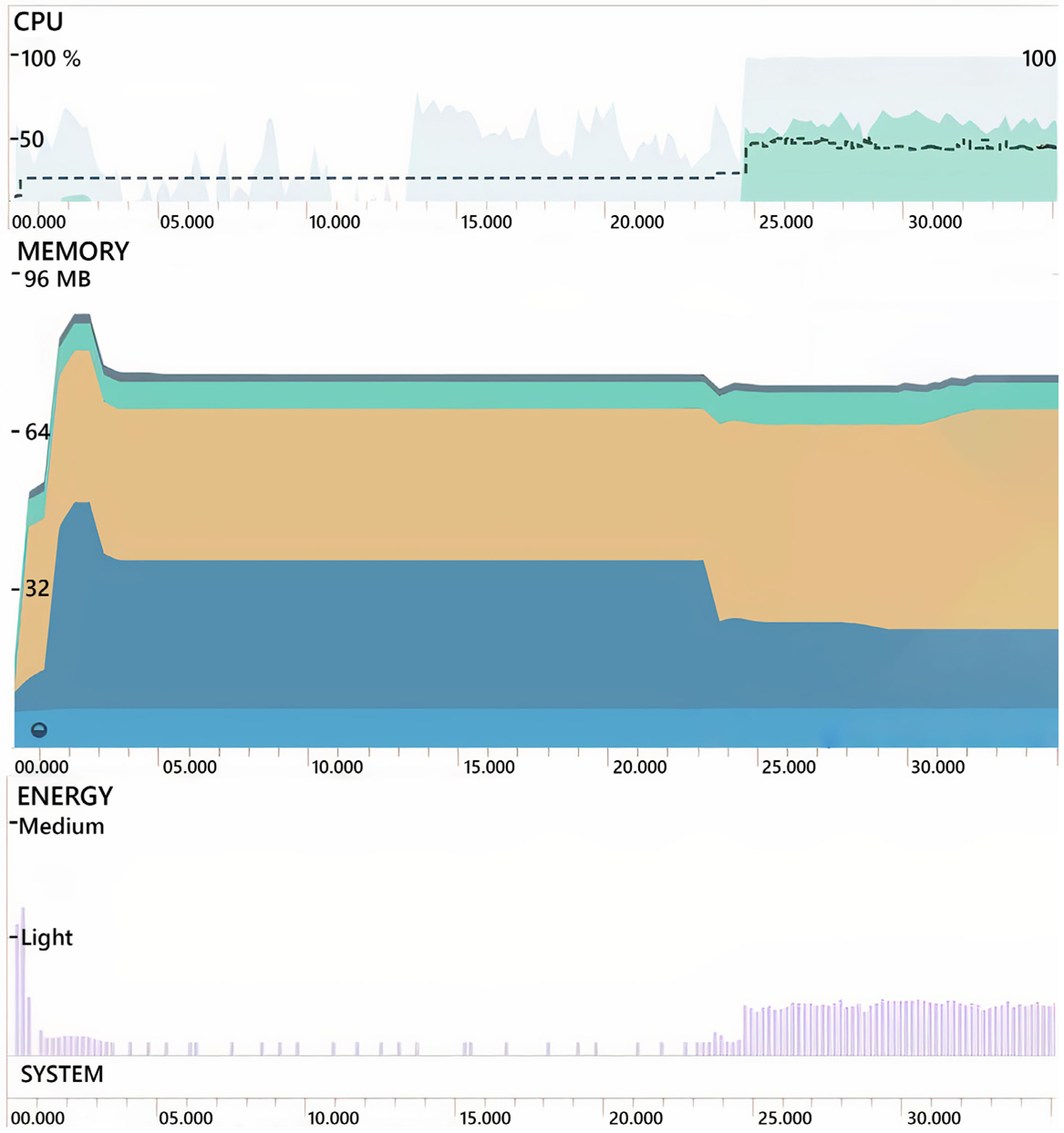
Snapshot of CPU (top), memory (middle) and energy (bottom) consumption of the proposed method on android pixel 3 smartphone.

The Central Processing Unit (CPU), memory, and energy usage of the application is also demonstrated in Figure 10 for the Pixel 3 smartphone. As it can be seen from Figure 10, the CPU usage of the app is around 50% when the application starts processing audio frames at 25th second. The memory utilization of the app after starting the application peaks at 88.8 MB and stabilizes around 74 MB after initializing a couple of frames. Modern smartphones in the market have a memory of a minimum of 4–6 GB, thus the memory consumption is quite low. These consumption results show that the app does not use massive CPU, memory, and energy resources of the smartphone. Additionally, the energy consumption is minimal, even though the CPU usage of the app is about 50%.

## VI. CONCLUSION

This paper presented a new approach for accurately localizing a sound source using especial L-shaped array with three microphones and its implementation on a Pixel 3 Android smartphone for hearing improvement. The proposed method uses an SF based VAD to improve the performance of the RSVD based DOA estimation. The work presented in this paper provides an optimized framework for real-time speech source localization using the three built-in microphones of a smartphone and demonstrates the achievement of real-time implementation of the proposed method on a smartphone under realistic noisy environments. The objective evaluation of the proposed method was analyzed and compared with other methods for different noise types at different SNRs. Analysis with recorded data shows that the real world conditions are more challenging due to the mixture of signal components in real environments. The highlighted framework was tested on a Pixel 3 smartphone with satisfactory results. The CPU, memory, and energy consumption of the proposed app were also evaluated. This method could also be extended with different VAD methods since the better classification of the incoming audio frames improves the performance of the system.

## Figures and Tables

**TABLE 1.** Summary table for recent works.

Algorithm	Methodology	Highlights and Limitations
Real-Time Estimation of Direction of Arrival of Speech Source using Three Microphones [29]	Time Delay Estimation (TDE)	Three microphone DOA approach using Generalized Cross Correlation. Improved performance under noise, but still lacks under very low SNR.
A TDOA-based multiple source localization using delay density maps [30]	TDE	This method focuses on multiple source localization using TDOA with volumetric mapping. The method was not examined with different noise types and low SNRs.
An L-shaped microphone array configuration for impulsive acoustic source localization in 2-D using orthogonal clustering based time delay estimation [54]	TDE	Utilizes orthogonal clustering algorithm for L-shaped microphone array. Only impulsive sources are considered without considering different SNRs.
Real-Time Convolutional Neural Network-Based Speech Source Localization on Smartphone [43]	Deep Learning	Convolutional Neural Network(CNN) approach for DOA. High accuracy but needs large dataset for training.
Multi-Speaker DOA Estimation Using Deep Convolutional Networks Trained With Noise Signals [47]	Deep Learning	CNN approach for multi-speaker DOA. Needs extensive data for model training.
A polynomial eigenvalue decomposition MUSIC approach for broadband sound source localization [35]	Multiple Signal Classification (MUSIC)	High resolution algorithm based on eigenvalue decomposition. Real time processing is not possible due to complexity.
DOA estimation of a system using MUSIC method [33]	MUSIC	Can be applied to different array geometries. The method is not able to identify of correlated signal and computationally complex.
A High-Accuracy, Low-Latency Technique for Talker Localization in Reverberant Environments Using Microphone Arrays [37]	Steered Response Power	Robust in noisy environment but has excessive computation due to the grid search.
Non-Uniform Microphone Arrays for Robust Speech Source Localization for Smartphone-Assisted Hearing Aid Devices [55]	Singular Value Decomposition	High performance under low SNR. Computationally very complex.

**TABLE 2.** Comparison of processing times for different data lengths.

	Processing Times			
L(Data Length)	MUSIC	SRP-PHAT	NU-SSL	Proposed
20 ms	49.8 ms	98.1 ms	9.7 ms	2.8 ms
100 ms	71.7 ms	537.5 ms	23.3 ms	5.5 ms
500 ms	97.2 ms	2914 ms	47.1 ms	12.7 ms

**TABLE 3.** RMSE(°) results for different angles.

	Angles					
SNR	0°	60°	120°	180°	240°	300°
−5 dB	6.46	4.92	5.07	6.12	5.39	5.86
0 dB	4.17	2.94	3.91	4.40	3.78	3.78
5 dB	2.5	1.96	1.75	2.29	1.64	1.63

## References

[1] (3. 1, 2020). Deafness and Hearing Loss. Accessed: Jun. 26, 2020. [Online]. Available: https://www.who.int/news-room/fact-sheets/detail/deafness-and-hearing-loss

[2] National Institute on Deafness and Other Communication Disorders. (12. 15, 2016). Quick Statistics About Hearing|NIDCD. Accessed: Jun. 26, 2020. [Online]. Available: https://www.nidcd.nih.gov/health/statistics/quick-statistics-hearing

[3] Types of Hearing Aids—Find the Right Hearing Aid for You. Accessed: Jun. 26, 2020. [Online]. Available: http://www.starkey.com/hearing-aids

[4] Oticon. (2018). Hearing Aids and Accessories for Any Hearing Loss|Oticon. accessed: Jun. 26, 2020. [Online]. Available: https://www.oticon.com/solutions

[5] Hearing Aids|Phonak. Accessed: Jun. 26, 2020. [Online]. Available: https://www.phonak.com/us/en/hearing-aids.html

[6] Van den Bogaert T, Doclo S, Wouters J, and Moonen M, “Speech enhancement with multichannel Wiener filter techniques in multimicro-phone binaural hearing aids,” J. Acoust. Soc. Amer, vol. 125, no. 1, pp. 360–371, 1. 2009, doi: 10.1121/1.3023069. 19173423

[7] Bhat GS, Shankar N, Reddy CKA, and Panahi IMS, “A real-time convolutional neural network based speech enhancement for hearing impaired listeners using smartphone,” IEEE Access, vol. 7, pp. 78421–78433, 2019, doi: 10.1109/ACCESS.2019.2922370. 32661495 PMC7357966

[8] Reddy CKA, Hao Y, and Panahi I, “Two microphones spectral-coherence based speech enhancement for hearing aids using smartphone as an assistive device,” in Proc. 38th Annu. Int. Conf. Eng. Med. Biol. Soc. (EMBC), Orlando, FL, USA, 8. 2016, pp. 3670–3673, doi: 10.1109/EMBC.2016.7591524. 28269090

[9] van Waterschoot T and Moonen M, “Fifty years of acoustic feedback control: State of the art and future challenges,” Proc. IEEE, vol. 99, no. 2, pp. 288–327, 2. 2011, doi: 10.1109/JPROC.2010.2090998.

[10] Khoubrouy SA, Panahi IMS, and Hansen JHL, “Howling detection in hearing aids based on generalized Teager–Kaiser operator,” IEEE Trans. Audio, Speech, Language Process, vol. 23, no. 1, pp. 154–161, 1. 2015, doi: 10.1109/TASLP.2014.2377575.

[11] Brandstein MS, “A framework for speech source localization using sensor arrays,” Brown Univ., Ann Arbor, MI, USA, Tech. Rep 9540732, 1995.

[12] McCowan I, “‘Microphone arrays: A tutorial,” Queensland Univ., Brisbane, QLD, Australia, Tech. Rep 4072, 2001, pp. 1–38.

[13] Ganguly A, Reddy C, Hao Y, and Panahi I, “Improving sound localization for hearing aid devices using smartphone assisted technology,” in Proc. IEEE Int. Workshop Signal Process. Syst. (SiPS), Dallas, TX, USA, 8. 2016, pp. 165–170, doi: 10.1109/SiPS.2016.37.

[14] Ganguly A, Kucuk A, and Panahi I, “Real-time Smartphone implementation of noise-robust speech source localization algorithm for hearing aid users,” in Proc. Meetings Acoust, vol. 30, no. 1, 2017, Art. no. 055002, doi: 10.1121/2.0000579.

[15] Kehtarnavaz N and Panahi IM, “Smartphones as research platform for hearing improvement studies,” J. Acoust. Soc. Amer, vol. 141, no. 5, p. 3495, 2017, doi: 10.1121/1.4987304.

[16] McCowan IA, Pelecanos J, and Sridharan S, “Robust speaker recognition using microphone arrays,” in Proc. Speaker Recognit. Workshop, 2001, pp. 101–106.

[17] Seltzer ML, Raj B, and Stern RM, “Speech recognizer-based microphone array processing for robust hands-free speech recognition,” in Proc. IEEE Int. Conf. Acoust., Speech, Signal Process, 2002, pp. I-897–I-900, doi: 10.1109/ICASSP.2002.5743884.

[18] Bronkhorst AW and Plomp R, “The effect of head-induced interaural time and level differences on speech intelligibility in noise,” J. Acoust. Soc. Amer, vol. 83, no. 4, pp. 1508–1516, 4. 1988, doi: 10.1121/1.395906. 3372866

[19] Bronkhorst AW and Plomp R, “Binaural speech intelligibility in noise for hearing-impaired listeners,” J. Acoust. Soc. Amer, vol. 86, no. 4, pp. 1374–1383, 10. 1989, doi: 10.1121/1.398697. 2808911

[20] Kuhn GF, “Model for the interaural time differences in the azimuthal plane,” J. Acoust. Soc. Amer, vol. 62, no. 1, pp. 157–167, 7. 1977, doi: 10.1121/1.381498.

[21] Wightman FL and Kistler DJ, “The dominant role of low-frequency interaural time differences in sound localization,” J. Acoust. Soc. Amer, vol. 91, no. 3, pp. 1648–1661, 3. 1992, doi: 10.1121/1.402445. 1564201

[22] Byrne D, Noble W, and LePage B, “Effects of long-term bilateral and unilateral fitting of different hearing aid types on the ability to locate sounds,” J. Amer. Acad. Audiol, vol. 3, no. 6, pp. 369–382, 1992. 1486199

[23] Keidser G, Rohrseitz K, Dillon H, Hamacher V, Carter L, Rass U, and Convery E, “The effect of multi-channel wide dynamic range compression, noise reduction, and the directional microphone on horizontal localization performance in hearing aid wearers,” Int. J. Audiol, vol. 45, no. 10, pp. 563–579, 1. 2006, doi: 10.1080/14992020600920804. 17062498

[24] Van den Bogaert T, Klasen T, Moonen M, Van Deun L, and Wouters J, “Horizontal localization with bilateral hearing aids: Without is better than with,” J. Acoust. Soc. Amer, vol. 119, no. 1, pp. 515–526, 1. 2006, doi: 10.1121/1.2139653. 16454305

[25] Noble W, Byrne D, and Lepage B, “Effects on sound localization of configuration and type of hearing impairment,” J. Acoust. Soc. Amer, vol. 95, no. 2, pp. 992–1005, 2. 1994, doi: 10.1121/1.408404. 8132913

[26] Brandstein MS and Griebel SM, “Nonlinear, model-based microphone array speech enhancement,” in Acoustic Signal Processing for Telecommunication. Boston, MA, USA: Springer, 2000, pp. 261–279.

[27] Zhang W and Rao BD, “A two microphone-based approach for source localization of multiple speech sources,” IEEE Trans. Audio, Speech, Language Process, vol. 18, no. 8, pp. 1913–1928, 11. 2010, doi: 10.1109/TASL.2010.2040525.

[28] Knapp CH and Carter GC, “The generalized correlation method for estimation of time delay,” IEEE Trans. Acoust., Speech Signal Process, vol. ASSP-24, no. 4, pp. 320–327, 8. 1976, doi: 10.1109/TASSP.1976.1162830.

[29] Tokgoz S, Kovalyov A, and Panahi I, “Real-time estimation of direction of arrival of speech source using three microphones,” in Proc. IEEE Workshop Signal Process. Syst. Implement, 10. 2020, pp. 1–5 doi: 10.1109/SIPS50750.2020.9195217. PMC8136617 34026330

[30] Boora R and Dhull SK, “A TDOA-based multiple source localization using delay density maps,” Sādhanā, vol. 45, no. 1, pp. 1–12, 8. 2020, doi: 10.1007/S12046-020-01453-8.

[31] Ganguly A, Kucuk A, and Panahi I, “Real-time smartphone application for improving spatial awareness of hearing assistive devices,” in Proc. 40th Annu. Int. Conf. Eng. Med. Biol. Soc. (EMBC), Honolulu, HI, USA, 7. 2018, pp. 433–436, doi: 10.1109/EMBC.2018.8512318. 30440427

[32] Schmidt R, “Multiple emitter location and signal parameter estimation,” IEEE Trans. Antennas Propag, vol. AP-34, no. 3, pp. 276–280, 3. 1986, doi: 10.1109/tap.1986.1143830.

[33] Devendra M and Manjunathachari K, “DOA estimation of a system using MUSIC method,” in Proc. Int. Conf. Signal Process. Commun. Eng. Syst, Guntur, India, 1. 2015, pp. 309–313, doi: 10.1109/SPACES.2015.7058272.

[34] Birnie LI, Abhayapala TD, and Samarasinghe PN, “Reflection assisted sound source localization through a harmonic domain MUSIC framework,” IEEE/ACM Trans. Audio, Speech, Lang. Process, vol. 28, pp. 279–293, 2020, doi: 10.1109/TASLP.2019.2953000.

[35] Hogg A, Vincent W, Weiss S, Evers C, and Naylor P, “A polynomial eigenvalue decomposition MUSIC approach for broadband sound source localization,” in Proc. IEEE Workshop Appl. Signal Process. Audio Acoust. (WASPAA), 12. 2021, p. 5.

[36] Roy R, Paulraj A, and Kailath T, “Direction-of-arrival estimation by subspace rotation methods–ESPRIT,” in Proc. IEEE Int. Conf. Acoust., Speech, Signal Process, 4. 1986, pp. 2495–2498.

[37] Dibiase JH, “A high-accuracy, low-latency technique for talker localization in reverberant environments using microphone arrays,” Brown Univ., Tech. Rep, 2000.

[38] Dmochowski JP, Benesty J, and Affes S, “A generalized steered response power method for computationally viable source localization,” IEEE Trans. Audio, Speech, Language Process, vol. 15, no. 8, pp. 2510–2526, 11. 2007, doi: 10.1109/TASL.2007.906694.

[39] Zotkin DN and Duraiswami R, “Accelerated speech source localization via a hierarchical search of steered response power,” IEEE Trans. Speech Audio Process, vol. 12, no. 5, pp. 499–508, 9. 2004, doi: 10.1109/TSA.2004.832990.

[40] Cedervall M and Moses RL, “Efficient maximum likelihood DOA estimation for signals with known waveforms in the presence of multipath,” IEEE Trans. Signal Process, vol. 45, no. 3, pp. 808–811, 3. 1997, doi: 10.1109/78.558512.

[41] Vorobyov SA, Gershman AB, and Wong KM, “Maximum likelihood direction-of-arrival estimation in unknown noise fields using sparse sensor arrays,” IEEE Trans. Signal Process, vol. 53, no. 1, pp. 34–43, 1. 2005, doi: 10.1109/TSP.2004.838966.

[42] Malioutov D, Çetin M, and Willsky AS, “A sparse signal reconstruction perspective for source localization with sensor arrays,” IEEE Trans. Signal Process, vol. 53, no. 8, pp. 3010–3022, 8. 2005, doi: 10.1109/TSP.2005.850882.

[43] Kucuk A, Ganguly A, Hao Y, and Panahi IMS, “Real-time convolutional neural network-based speech source localization on smartphone,” IEEE Access, vol. 7, pp. 169969–169978, 2019, doi: 10.1109/ACCESS.2019.2955049. 32754421 PMC7402615

[44] Sun Y, Chen J, Yuen C, and Rahardja S, “Indoor sound source localization with probabilistic neural network,” IEEE Trans. Ind. Electron, vol. 65, no. 8, pp. 6403–6413, 8. 2018, doi: 10.1109/TIE.2017.2786219.

[45] Pertila P and Parviainen M, “Time difference of arrival estimation of speech signals using deep neural networks with integrated time-frequency masking,” in Proc. IEEE Int. Conf. Acoust., Speech Signal Process. (ICASSP), Brighton, U.K., 5 2019, pp. 436–440, doi: 10.1109/ICASSP.2019.8682574.

[46] Vecchiotti P, Ma N, Squartini S, and Brown GJ, “End-to-end binaural sound localisation from the raw waveform,” in Proc. IEEE Int. Conf. Acoust., Speech Signal Process. (ICASSP), Brighton, U.K., 5 2019, pp. 451–455, doi: 10.1109/ICASSP.2019.8683732.

[47] Chakrabarty S and Habets EAP, “Multi-speaker DOA estimation using deep convolutional networks trained with noise signals,” IEEE J. Sel. Topics Signal Process, vol. 13, no. 1, pp. 8–21, 3. 2019, doi: 10.1109/JSTSP.2019.2901664.

[48] Tashev IJ, Sound Capture and Processing: Practical Approaches. Hoboken, NJ, USA: Wiley, 2009.

[49] Brandstein M and Ward D, Eds., Microphone Arrays-Signal Processing Techniques and Applications. Berlin, Germany: Springer, 2001.

[50] Desai D and Mehendale N, “A review on sound source localization systems,” SSRN Electron. J, 7. 2021. [Online]. Available: https://papers.ssrn.com/sol3/papers.cfm?abstract_id=3891373, doi: 10.2139/ssrn.3891373.

[51] Liaquat MU, Munawar HS, Rahman A, Qadir Z, Kouzani AZ, and Mahmud MAP, “Localization of sound sources: A systematic review,” Energies, vol. 14, no. 13, p. 3910, 6. 2021, doi: 10.3390/en14133910.

[52] Widrow B and Luo F-L, “Microphone arrays for hearing aids: An overview,” Speech Commun, vol. 39, nos. 1–2, pp. 139–146, 2003, doi: 10.1016/S0167-6393(02)00063-8.

[53] Tellakula AK, “Acoustic source localization using time delay estimation,” Degree thesis, Supercomputer Educ. Res. Centre, Indian Inst. Sci, Bangalore, India, 2007.

[54] Omer M, Quadeer AA, Al-Naffouri TY, and Sharawi MS, “An L-shaped microphone array configuration for impulsive acoustic source localization in 2-D using orthogonal clustering based time delay estimation,” in Proc. 1st Int. Conf. Commun., Signal Process., Appl. (ICCSPA), 2. 2013, pp. 1–6, doi: 10.1109/ICCSPA.2013.6487241.

[55] Ganguly A and Panahi I, “Non-uniform microphone arrays for robust speech source localization for smartphone-assisted hearing aid devices,” J. Signal Process. Syst, vol. 90, no. 10, pp. 1415–1435, 10. 2018, doi: 10.1007/s11265-017-1297-8. 30294408 PMC6168089

[56] Sadjadi SO and Hansen JHL, “Unsupervised speech activity detection using voicing measures and perceptual spectral flux,” IEEE Signal Process. Lett, vol. 20, no. 3, pp. 197–200, 3. 2013, doi: 10.1109/LSP.2013.2237903.

[57] Ganguly A, “Noise-robust speech source localization and tracking using microphone arrays for smartphone-assisted hearing aid devices,” Ph.D. dissertation, Univ. Texas Dallas, 2018. 10.1007/s11265-017-1297-8 PMC6168089 30294408

[58] Halko N, Martinsson PG, and Tropp JA, “Finding structure with randomness: Probabilistic algorithms for constructing approximate matrix decompositions,” SIAM Rev, vol. 53, no. 2, pp. 217–288, 12. 2011, doi: 10.1137/090771806.

[59] Feng X, Yu W, and Li Y, “Faster matrix completion using randomized SVD,” in Proc. 30th Int. Conf. Tools Artif. Intell. (ICTAI), 11. 2018, pp. 608–615, doi: 10.1109/ICTAI.2018.00098.

[60] Sohn J, Kim NS, and Sung W, “A statistical model-based voice activity detection,” IEEE Signal Process. Lett, vol. 6, no. 1, pp. 1–3, 1. 1999, doi: 10.1109/97.736233.

[61] Garofolo JS, Lamel LF, Fisher WM, Fiscus JG, Pallett DS, Dahlgren NL, and Zue V, TIMIT Acoustic-Phonetic Continuous Speech Corpus LDC93S1. Philadelphia, PA, USA: Linguistic Data Consortium, 1993.

[62] Nilsson M, Soli SD, and Sullivan JA, “Development of the Hearing in Noise Test for the measurement of speech reception thresholds in quiet and in noise,” J. Acoust. Soc. Amer, vol. 95, no. 2, pp. 1085–1099, 1994, doi: 10.1121/1.408469. 8132902

[63] Lehmann EA and Johansson AM, “Diffuse reverberation model for efficient image-source simulation of room impulse responses,” IEEE Trans. Audio, Speech, Language Process, vol. 18, no. 6, pp. 1429–1439, 8. 2010, doi: 10.1109/TASL.2009.2035038.

[64] Smartphone-Based Open Research Platform for Hearing Improvement Studies. Accessed: Sep. 29, 2020. [Online]. Available: https://labs.utdallas.edu/ssprl/hearing-aid-project/

